# Macular irregularities of optical coherence tomographic vertical cross sectional images in school age children

**DOI:** 10.1038/s41598-021-84808-3

**Published:** 2021-03-05

**Authors:** Takehiro Yamashita, Hiroto Terasaki, Ryo Asaoka, Naoya Yoshihara, Naoko Kakiuchi, Taiji Sakamoto

**Affiliations:** 1 Department of Ophthalmology, Kagoshima University Graduate School of Medical and Dental Sciences, Kagoshima, Japan; 2 Department of Ophthalmology, Seirei Hamamatsu General Hospital, Hamamatsu, Shizuoka Japan; 3 Seirei Christopher University, Hamamatsu, Shizuoka Japan

**Keywords:** Anatomy, Medical research

## Abstract

The purpose of this study was to compare the incidences of macular irregularities of elementary school (ES) and junior high school (JHS) students. This was a prospective cross-sectional observational study of 122 right eyes of 122 ES students (8–9 years) and 173 right eyes of 173 JHS students (12–13 years). Vertical cross-sectional images of the macula were obtained by optical coherence tomography. The eyes were classified based on the vertical symmetry of the posterior pole, and then sub-classified as convex-, flat-, concave-, or dome-shaped based on the direction of the curvature of the retinal pigment epithelium. One hundred and two eyes (83.6%) were placed in the symmetrical group in the ES students and 149 eyes (86.1%) in the JHS students. Twenty eyes (16.4%) were placed in the asymmetric groups in the ES students and 24 eyes (13.9%) in the JHS students. In symmetrical group, 78 and 118 eyes had a convex-shape (76.4 and 79.2%), followed by 22 and 29 eyes of dome-shaped group (21.6 and 19.4%) in ES and JHS students respectively. Because the incidences of the posterior pole shapes were not significantly different between the groups, it is likely that the macular irregularities develop before the age of ES.

## Introduction

A dome-shaped macula (DSM) is an inward protrusion of the macula that can be seen in the optical coherence tomographic (OCT) images^1–3^. Earlier studies have shown that some of the eyes with DSMs had changes called irregular shaped macula including Bruch's membrane defects, myopic choroidal neovascularization-related macular atrophy, myopic choroidal atrophy, and serous retinal detachment^4–10^.

An inferior staphyloma is an outward protrusion of the inferior macula as seen in OCT images^11–13^. It is also associated with irregularities in the shape of the macula including the tilted disc syndrome, focal retinal thinning, choroidal neovascularization, and serous retinal detachment^14–21^. Because these irregularities are progressive, it is important to detect them at an early phase.

An earlier study showed that macular irregularities, such as DSMs and inferior staphylomas, can already be present in the highly myopic eyes of children^10^. On the other hand, there are many adult patients with macular irregularities in non-highly myopic eyes^22^. We hypothesized that there can be small-scale macular irregularities already in eyes of normal children.

Currently, it is necessary to magnify the OCT images of the retina to make more accurate findings of macular irregularities. A 2X magnification in the anterior–posterior axis of the OCT displayed images has been used to examine the structural changes of the macular area even though the images are distorted. This helped in detecting the irregular shapes of the posterior pole of the eye such as the DSMs and inferior staphylomas^23,24^. We have recently developed a method to detect the irregular structures of the macula by magnifying the displayed image 8X in the anterior–posterior direction which improved the sensitivity of detecting abnormal shapes of the posterior pole of the eye. We found that even slight changes in the structural configuration of the macula were significantly associated with eyes with glaucoma^22^.

It has not been determined when the irregular shapes of the macula, such as the DSMs and inferior staphylomas, develop. Xu et al. reported elevated maculas in highly myopic eyes of children and ridge-shaped macula in adolescents indicating that these shapes can already be present in childhood^10^. However, their study population were those with high myopia which might be a unique population. To the best of our knowledge, there is no report on the incidence of irregular shaped posterior pole of the eyes of healthy children.

Thus, the purpose of this study was to compare the incidence of irregular shapes of the macular region of the eyes of elementary school (ES) and junior high school (JHS) students.

## Results

The mean AL was 23.39 ± 0.90 mm with a range from 20.52 to 25.80 mm in the ES students consisting of 61 boys and 61 girls, and 24.57 ± 1.21 mm with a range from 21.91 to 28.94 mm in the JHS student consisting of 83 boys and 90 girls. The mean AL of the JHS students was significantly longer than that of ES students (*P* < 0.001; Mann–Whitney U test).

In the ES students, 102 (84%) eyes were placed in the symmetrical group, and 78 of these were place in the convex-shaped group, 2 eyes in the flat-shaped group, 0 eyes in the concave-shaped, and 22 eyes in the dome-shaped group. Twenty eyes (16%) were placed in the asymmetric group of which 7 eyes were placed in the convex-shaped group, 4 eyes in the flat-shaped group, 2 eyes in the concave-shaped group, and 7 eyes in the dome-shaped group in the superior half or the retina. In the inferior retina, 5 eyes were placed in the convex-shaped group, 2 eyes in the flat-shaped group, 8 eyes in concave-shaped group, and 5 eyes in the dome-shaped group (Table 1).

**Table 1:** Macular morphology in elementary/junior high school students.

Symmetry (bold) Asymmetry (white)		Upper part			
		Convex	Flat	Concave	Dome
Lower part	Convex	**78/118**	3/0	0/0	2/1
	Flat	1/0	**2/1**	1/0	0/1
	Concave	2/3	1/0	**0/1**	5/4
	Dome	4/14	0/1	1/0	**22/29**

In the JHS students, 149 eyes (86%) were placed in the symmetric group of which 118 eyes were place in the convex-shaped group, 1 eye in the flat-shaped group, 1 eye in the concave-shaped group, and 29 eyes in the dome-shaped group. Twenty-four eyes (14%) were placed in the asymmetric group of which 17 eyes were placed in the convex-shaped group, 1 eye in the flat-shaped group, 0 eyes in the concave-shaped group, and 6 eyes in the dome-shaped group in the superior retina. In the inferior retina, 1 eye was placed in the convex-shaped group, 1 eye in the flat-shaped group, 7 eyes in the concave-shaped group, and 15 eyes in the dome-shaped group (Table 1).

The distribution of the eyes in the symmetrical eyes was not significantly different between the ES students and JHS students (*P* = 0.62; Fisher’s exact test). The distribution of the shapes of the macular in the symmetrical eyes was also not significantly different between the ES and JHS students (*P* = 0.74; Fisher’s exact test). There was no significant difference in the axial length between the symmetrical convex-shaped group (23.42 ± 0.86 mm) and the symmetrical dome-shaped group (23.57 ± 0.73 mm) in the ES students (*P* = 0.34; Mann–Whitney U test). The differences in the axial length between the symmetric convex (24.69 ± 1.24 mm) and symmetric dome (24.67 ± 1.05 mm) in the JHS students were also not significant (*P* = 0.94; Mann–Whitney U test).

## Discussion

The differences in the shape of the posterior pole of the eye such as the dome-shaped macula or the concave-shaped macula were observed in 38 of 122 eyes (31.1%) in the ES students. In JHS students, 54 of 173 eyes (31.2%) had similar differences in the shape of the posterior pole of the eyes. Thus, although the axial length of JHS students was significantly longer than that of ES students, the incidences of the different shapes of the maculae were not significantly different between the two groups of children of different ages. All of the students of the same school that met the inclusion criteria were studied except for those with no parental consent. In a strict sense, it is necessary to follow these cohorts longitudinally to evaluate the changes in the shape of the eye. However, it is possible to obtain a good estimation of the changes in the shape of the eyes by examining the different age groups in the same population. Thus, it is possible to say that the differences between these two age groups reflects the changes from elementary school age to junior school age. Similarly, this irregular shape of the posterior pole of the eye did not develop between 8 and 13 years but before 8-years-of-age.

It is difficult to explain this phenomenon just from the present observations. One possible explanation is that the choroidal non-vascular smooth muscle cells develop before 6-years-of-age^25^. The non-vascular smooth muscle cells are located between the choroid and sclera and they are richly distributed between the optic the nerve and macula and along the vortex veins. The non-vascular smooth muscle cells along the vortex veins play a role in autoregulation, while the role of those in the macular area are not known and their density is quite different in each eye^25^. In our earlier study, we hypothesized that these smooth muscle cells surrounding the macular area function to keep the macular area stable as the axial length increases^26^. The individual difference of these choroidal smooth muscles may affect the irregular shape of the posterior pole of the eye of school students.

To the best of our knowledge, there has not been a comparable study on the incidences of the irregular shapes of the posterior pole of the eye. However, we have studied the incidences of the irregular shapes in glaucomatous adult eyes using the same method. In glaucomatous eyes, 70 of the 83 eyes (84.3%) were classified into the symmetric group and 13 eyes (15.7%) were classified into the asymmetric group^22^. These values are comparable to those found in the ES and JHS students with 84% symmetrical and 16% asymmetric in ES students and 86% of the eyes were symmetrical and 14% were asymmetric in the JHS students. The dome-shaped macular was found in 22 of 122 eyes (18%) of ES students and in 29 of 149 eyes (19.5%) in JHS students. In glaucomatous eyes, 6 of the 83 eyes (7.2%) were classified with DSM^22^. These results suggest that the incidence of the DSM does not increase during this period. The other possibility is that eyes with DSM in glaucomatous eyes. Those with lower concavity are supposed to be related to an inferior staphyloma which was found in 8 of 122 eyes (6.6%) in ES and 8 of 149 eyes (5.4%) in JHS students. Because the prevalence was higher in glaucomatous adult eyes (14.5%)^22^, the presence of an inferior staphyloma may be related to the presence of glaucoma.

Of interest was that there was no significant difference in the axial lengths between eyes in the convex-shaped group and the dome-shaped group. This suggests that the convex-shaped eyes would have longer axial lengths than dome-shaped eyes, but it was not the case either in the ES or the JHS students.

There are limitations in this study. The classification was done by examination of only a single fovea vertical section, and horizontal cross-sectional images were not examined. Although the examination of a single section was easily done, it does not always reflect the actual shape of the posterior pole of the eye^27^. Unfortunately, this additional examination was not allowed by the Ethic Committee because the examination time was required to be minimal for healthy school students. The second limitation was that these are the results of Japanese population which is the most myopic population in the world^28^. Thus, the results may not be generalized to other populations. A third limitation was this was a cross-sectional study. To determine the effect of growth, a longitudinal study is necessary. In fact, we are performing a longitudinal study of the present groups which should provide information on the changes in the incidences of the macular shapes. The fourth limitation was that we detected the subtle changes of posterior eye structure by magnifying the vertical axis 8X. Thus, the results may not be comparable to those of earlier studies in which the vertical images were magnified 2X.

In conclusion, there is already differences in the shape of the posterior pole of the eyes in ES and JHS students. During this period, there were no significant changes in the incidences of the different macular shapes. These findings should help researchers investigate the relationship of the macular shapes and glaucoma or other ophthalmic diseases.

## Methods

### Ethics statement

All of the procedures used in this study conformed to the tenets of the Declaration of Helsinki, and they were approved by the Ethics Committee of Kagoshima University Hospital. A written informed assent and informed consent were obtained from all of the subjects and their parents. A written informed consent for publication of clinical images were also obtained from the subjects and their parents. This study was registered with the University Hospital Medical Network-clinical trials registry (No. UMIN000015239).

### Subjects

This was a prospective, cross sectional, observational study performed on 8- to 9-year old third grade students of an ES and 12- to 13-year-old first grade students of a JHS of the Faculty of Education of Kagoshima University^29,30^.

There were 144 students in the ES group and 200 students in the JHS group. An informed consent obtained from 122 (87.4%) students and their parents in the ES group and 178 (89.0%) students and their parents in the JHS group. Students in the ES group were examined from November 17 to December 18, 2014, and those in the JHS group from January 13 to February 13, 2015. Vertical cross-sectional images of macula and color fundus photographs were taken with the 3D OCT-1 Maestro OCT device (Topcon, Tokyo, Japan). The axial length was measured with the OA-2000 Optical Biometer (Tomey, Nagoya, Japan). Only the right eyes were measured to avoid false confidence intervals and low *P* values. Five eyes were excluded due to the presence of ocular disorders; three cases of superior segmental optic hypoplasia and two cases of optic nerve atrophy. In the end, the right eyes of 122 ES students and 173 JHS students were used for the statistical analyses.

### Optical coherence tomographic (OCT) measurements

OCT images were obtained with the 3D OCT-1 Maestro (Topcon, Tokyo, Japan) with the 3D macular vertical raster scan mode and 6 × 6 mm scan area. This device operates at a speed of 50 000 A-scans/second and has a depth resolution of 6 mµ and a lateral resolution of 20 µm. All OCT recordings were made without pupillary dilation. The ocular magnification was automatically adjusted based on the axial length. The exclusion criterion for the OCT analyses was an image quality index of < 30.

### Classification of macular shape

**Figure 1:**
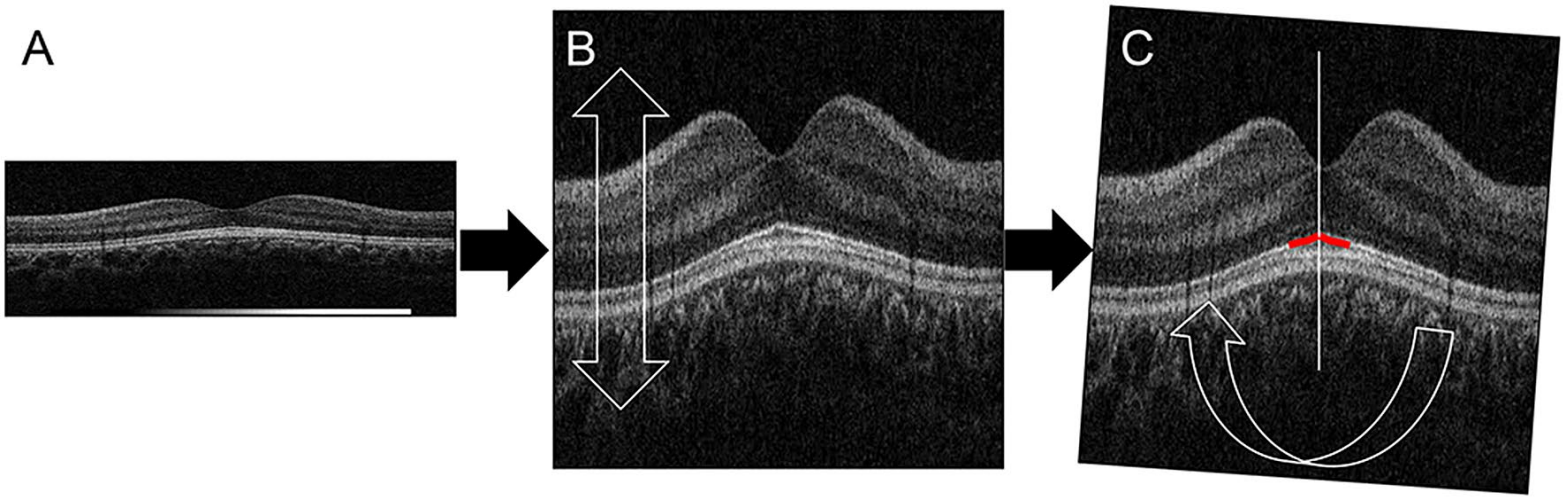
Magnification of the optical coherence tomographic (OCT) vertical cross-sectional image. First, the original 2X B-scan image (**A**) was vertically expanded 4X (**B**, vertical-to-horizontal ratio, 8:1) for easier detection of the macular shape. Second, the image was rotated to make the ellipsoid zone around the fovea symmetrical (**C**).

A vertical cross-sectional image of the fovea that was obtained using the 3D macular vertical raster scan mode was used to classify the macular shape. In our preliminary study, we examined the OCT images of 10 pediatric eyes and evaluated different axial magnifications of the images by 2X, 4X, 6X, 8X, 10X, and 12X. We concluded that 8X was most suitable for the examination by our committee (TY, HT, and TS). So 8X was used throughout the study. First, the displayed original 2X B-scan image was vertically magnified 4X (vertical-to-horizontal ratio, 8:1) to examine the macular morphology in greater detail. Second, the image was rotated to make the ellipsoid zone around the fovea symmetrical. Third, the image was classified by its shape based on its vertical symmetry, and then sub-classified into convex-shaped, flat-shaped, concave-shaped, and dome-shaped according to the curvature of the retinal pigment epithelium (RPE) as we reported in detail (Fig. 1)^22^.

**Figure 2:**
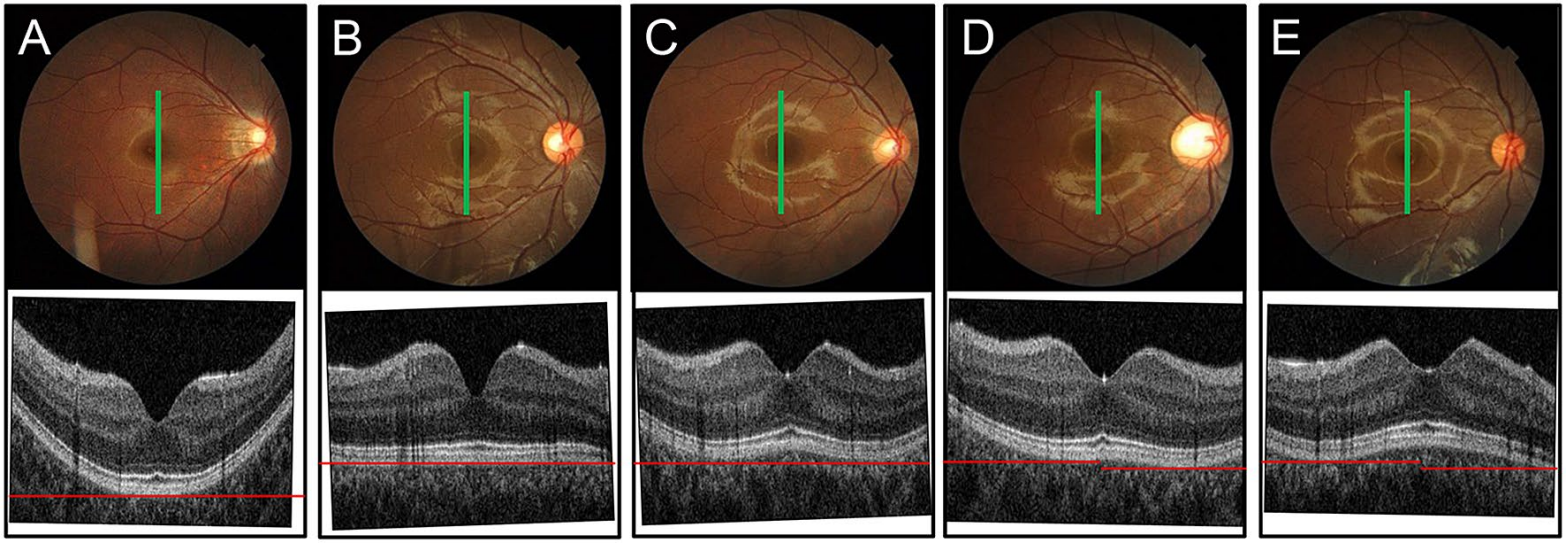
Classification of shapes of macular vertical cross-sectional images. The OCT images were classified based upon its vertical symmetry. Images in (**A**, **B**), and (**C**) are symmetrical, and images in (**D**) and (**E**) are asymmetrical. The images are then sub-classified into convex-shaped (**A**, **D** upper part), flat-shaped (**B**), dome-shaped (**C**, **D** lower part, **E** upper part), and concave-shaped (**E** lower part) according to the retinal pigment epithelium curvature. Red lines are horizontal straight lines.

To make this procedure objective, a straight line was placed beneath the highly reflective RPE line and the following criteria were used (Fig. 2). First, the OCT images were classified based upon its vertical symmetry. Images in Fig. 2A–C are symmetrical, and images in Fig. 2D–E are asymmetrical. The “convex-shaped” type of maculae were those whose center sat on the straight line and the inferior and superior edges of the RPE were curved toward the interior of the eye (Fig. 2A, D upper part). A “flat” type macula was classified for eyes whose shape of the RPE in the OCT image was flat from the superior and inferior edges to the fovea. In these flat-shaped eyes, the total RPE could be fit by a straight line (Fig. 2B). A “dome-shaped” macula was classified for the eyes in which the RPE under the fovea was concave toward the interior of the eye and the shape of the RPE between the fovea and the superior or inferior edge was concave toward the exterior of the eye. Thus, the center of RPE is over the straight line and peripheral RPE is attached to the line at two points and it looks like a letter ‘W’. (Fig. 2C, D lower part, E upper part). The “concave-shaped” type of maculae were those in which the center of the RPE on the image was attached to the straight line at the edge of the line. (Fig. 2E lower part). The classification and sub-classification were manually done for each image independently by 2 graders (NY and TY). When the results of the 2 graders did not agree, the final classification and subclassification were decided by an examination and discussion with a third grader (HT).

### Statistical analyses

All statistical analyses were performed with the statistical programming language R (version 3.0.2, The R Foundation for Statistical Computing, Vienna, Austria). Fisher’s exact tests were used to determine the significant differences in the incidences of the different macular shapes between the ES and JHS groups. Mann–Whitney U tests were used to determine the significance of the differences in the axial lengths between the groups.
